# Congestive Nephropathy: A Neglected Entity in Heart Failure

**DOI:** 10.7759/cureus.94211

**Published:** 2025-10-09

**Authors:** Muammer Avci, Fatma Avci Merdin, Ela Güven Avci

**Affiliations:** 1 Nephrology, Isparta City Hospital, Republic of Turkey Ministry of Health, Isparta, TUR; 2 Endocrinology and Metabolism, Isparta City Hospital, Republic of Turkey Ministry of Health, Isparta, TUR; 3 Geriatrics, Faculty of Medicine, Süleyman Demirel University, Isparta, TUR

**Keywords:** cardiorenal syndrome, congestive nephropathy, fluid challenge test, heart failure without hypervolemia, heart failure without weight gain, pocus (point of care ultrasound), venous reservoir capacitance

## Abstract

Contrary to common belief, the primary cause of acute kidney injury in patients presenting to emergency departments with heart failure is not diminished renal perfusion but rather renal venous congestion and increased pressure. In patients without overt signs of heart failure during initial physical assessment, however, congestion often cannot be distinguished through physical examination alone, leading to congestive nephropathy being frequently overlooked in the differential diagnosis of worsening renal function. Physicians often avoid diuretics for fear of exacerbating renal impairment and instead opt for fluid replacement. This approach can further deteriorate renal function.

We describe the clinical course of a patient with heart failure and impaired renal function whose diagnosis of congestive nephropathy was initially overlooked. A 68-year-old male with a history of heart failure, pulmonary hypertension, and chronic obstructive pulmonary disease was admitted to the cardiovascular surgery service for deep vein thrombosis and acute kidney injury. His physical examination upon admission revealed a blood pressure of 135/85 mmHg. Lung sounds were diminished in the basal zones, with expiratory rhonchi and wheezing in the mid-zones, consistent with his underlying pulmonary disease. The cardiac examination was unremarkable. The lower extremity affected by deep vein thrombosis was locally swollen and erythematous, while the other limb had no edema. His oral mucosa and the dorsum of his tongue were dry. With a creatinine level of 1.3 mg/dL (baseline: 0.9 mg/dL), his condition was assessed as prerenal acute kidney injury because the initial physical findings showed no evidence of hypervolemia; consequently, fluid replacement was administered. On the third day, after his renal function further deteriorated, a nephrology consultation was requested. At this time, his physical examination revealed prominent signs of hypervolemia and symptoms of heart failure. Intravenous furosemide was initiated on the fourth day. Renal function improved rapidly after discontinuing fluid replacement and initiating diuretic therapy. Differential diagnoses for patients with heart failure presenting with impaired renal function should include dehydration, as well as drug- and infection-related kidney injury. Congestive nephropathy can develop in all forms of heart failure and should therefore be considered in patients with unexplained renal dysfunction. Proper management of congestive nephropathy is associated with fewer long-term hospital readmissions for heart failure and improved survival rates. In ambiguous cases, the differential diagnosis can be established through point-of-care ultrasonography or, when unavailable, a diagnostic fluid challenge.

## Introduction

In patients presenting to the emergency department with heart failure, a primary driver of renal dysfunction is usually elevated renal venous pressure, a mechanism distinct from the long-held assumption of diminished arterial perfusion [1]. Impaired renal function is observed in approximately one in three patients admitted with acute heart failure [2]. This impairment is typically a reversible, hemodynamic, and functional condition [3]. The pathophysiology is multifactorial, but the traditional explanation posits that deteriorating systolic and diastolic function reduces cardiac output, which in turn activates the neurohormonal and sympathetic nervous systems [2]. This activation leads to systemic and renal vasoconstriction, diminishing renal perfusion and impairing kidney function [4]. According to this model, renal impairment is directly proportional to the reduction in blood pressure and renal perfusion [4]. However, a growing body of observational studies has identified renal venous congestion as a more significant pathophysiological factor in this patient population [5]. Acute heart failure can lead to right-sided heart failure, elevating right atrial and central venous pressures and causing renal venous congestion [5]. This process is often exacerbated by conditions, such as chronic obstructive pulmonary disease (COPD) and pulmonary hypertension [6]. As the kidney is an encapsulated organ with limited compliance, the increased venous pressure is transmitted to the efferent arteriole and the tubulointerstitial space [7]. This reduces the net glomerular filtration pressure, leading to a decrease in the estimated glomerular filtration rate (eGFR) [7]. Concurrently, heightened neurohormonal and sympathetic activity promotes sodium and water reabsorption, while worsening arteriolar vasoconstriction disrupts renal autoregulation, contributing to a further decline in eGFR [8]. This condition is defined as “congestive nephropathy” [5]. Overt congestion may not be detectable through traditional diagnostic methods, such as examination for jugular venous distention, an S3 heart sound, lower extremity edema, or findings on a chest X-ray [9]. This diagnostic challenge frequently leads to the omission of congestive nephropathy from the differential diagnosis of worsening renal function. The mainstay of treatment for congestive nephropathy is decongestion with high-dose diuretics; in cases of diuretic resistance, ultrafiltration should be considered [10]. Early and aggressive decongestion can improve ventricular filling and renal blood flow, reducing the progression of heart failure and thereby lowering hospital readmission and mortality rates [11]. Point-of-care ultrasonography (POCUS) is evolving as a more effective tool than traditional methods for identifying subclinical organ congestion [12]. An objective, POCUS-based diagnosis facilitates appropriate decongestive therapy, which is crucial for reducing the mortality and morbidity associated with unresolved congestion [11]. In this report, we present a case of a patient who initially presented to the emergency department with deep vein thrombosis and cellulitis, and we describe the clinical course following an overlooked diagnosis of congestive nephropathy.

## Case presentation

A 68-year-old male with a past medical history of chronic obstructive pulmonary disease (COPD), type 2 diabetes mellitus, hypertension, and heart failure was admitted to the cardiovascular surgery service. He presented with a one-week history of swelling and erythema in his left lower leg, leading to a diagnosis of deep vein thrombosis (DVT). The nephrology service was subsequently consulted for progressive dyspnea and worsening renal function. At the time of the nephrology consultation, his blood pressure was 135/85 mmHg. Lung auscultation revealed bilateral crackles extending to the mid-zones. He had +2 pitting edema in both lower extremities, and his left calf remained swollen and erythematous. The remainder of the physical examination was unremarkable. Laboratory studies indicated a serum creatinine of 2.36 mg/dL. An echocardiogram showed a preserved ejection fraction (EF) of 60%, dilated right atrial and ventricular chambers, and evidence of elevated right-sided pressures, including grade II tricuspid regurgitation and an estimated pulmonary artery pressure of 48 mmHg. Additional laboratory findings are detailed in Table 1.

**Table 1:** Laboratory values at presentation. MCV: mean cell volume; eGFR: estimated glomerular filtration rate; AST: aspartate aminotransferase; ALT: alanine aminotransferase; BUN: blood urea nitrogen; INR: international normalized ratio

Measurement (SI unit)	Value (laboratory normal range)
Hemoglobin (g/L)	116 (120-160)
MCV (fL)	65.2 (80-97)
Leukocytes (10^9^/L)	13.2 (4-11)
Neutrophils (10^9^/L)	11.5 (2-7.5)
Lymphocytes (10^9^/L)	0.76 (1.5-4.5)
Platelets (10^9^/L)	168 (150-400)
Glucose (mg/dL)	93 (74-106)
Sodium (mmol/L)	134 (136-146)
Potassium (mmol/L)	6.1 (3.5-5.5)
Chlorine (mmol/L)	104 (101-109)
BUN (mg/dL)	51 (8-20)
Creatinine (mg/dL)	1.3 (0.8-1.4)
eGFR (mL/min)	58
Albumin (g/L)	38.7 (30-50)
Calcium (mg/dL)	8.7 (8.8-10.6)
Phosphorus (mg/dL)	2.6 (2.5-4.5)
Magnesium (mg/dL)	2.1 (1.8-2.6)
AST (U/L)	14 (10-50)
ALT (U/L)	8 (0-50)
Alkaline phosphatase U/L	105 (30-130)
Total bilirubin (mg/dL)	1.2 (0.3-1.2)
Direct bilirubin (mg/dL)	0.59 (<0.2)
C-reactive protein (mg/dL)	81.8 (<5)
INR	1.08 (<1.1)
Prothrombin time (s)	12.8 (10-13)

**Figure 1:**
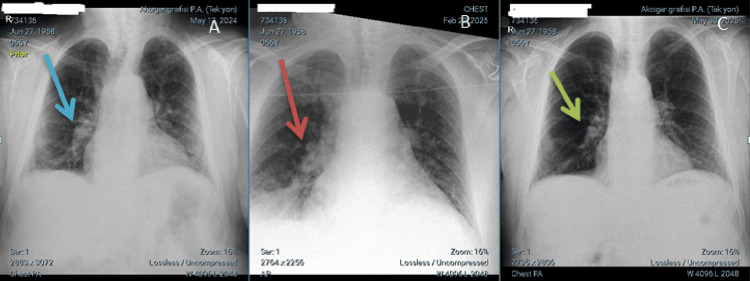
Chest radiographs from A to C obtained in outpatient clinic, consultation, and discharge, respectively. (A) The blue arrow indicates a dilated pulmonary artery in the patient with COPD. The cardiothoracic ratio is slightly increased, and the heart shadow is slightly enlarged. (B) The cardiothoracic ratio and cardiac shadows are enlarged. The red arrow indicates the dilated right main pulmonary artery and pulmonary edema. (C) The cardiothoracic ratio decreased, and the dimensions of the heart shadow returned to baseline. The green arrow shows a shrunken pulmonary artery. P.A.: posterior anterior; COPD: chronic obstructive pulmonary disease

A review of his hospital course revealed that upon his initial presentation three days prior, his creatinine was 1.3 mg/dL (patient’s baseline: 0.9 mg/dL) and potassium was 6.1 mmol/L. Upon presentation, his physical examination was notable for a swollen, erythematous left lower extremity without edema in the contralateral limb. Lung sounds were consistent with his history of COPD, with diminished sounds at the bases and expiratory rhonchi in the mid-zones. Figures 1A-1C show the patient's baseline chest X-ray obtained during an outpatient pulmonology follow-up, alongside those from his admission and postdischarge.

**Figure 2:**
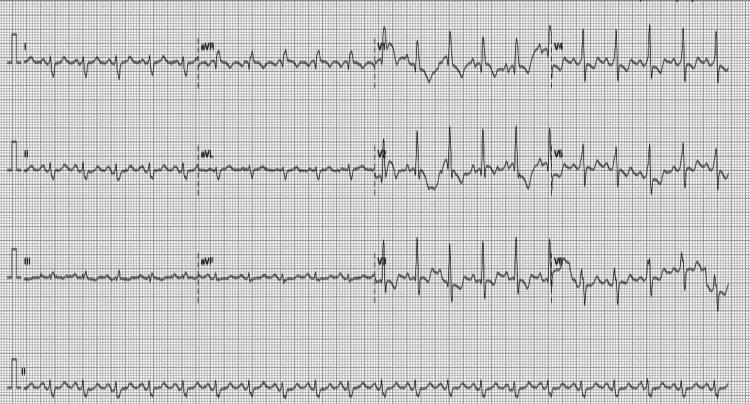
ECG obtained at the time of consultation. Sinus tachycardia, right axis shift, V1-4, and right ventricular strain pattern with T wave inversion.

**Figure 3:**
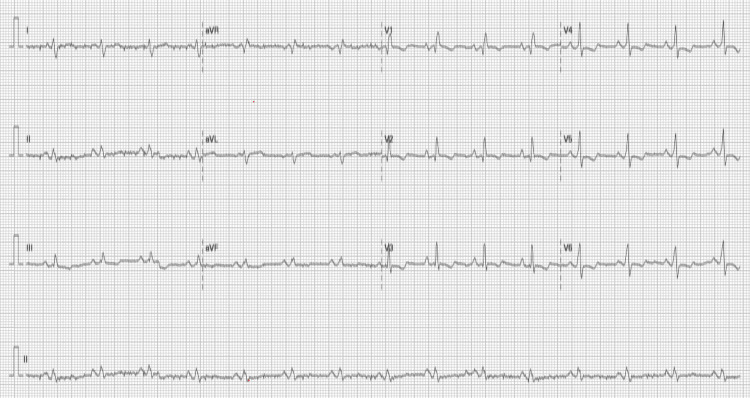
ECG obtained at the time of renal recovery. Sinus rhythm, right axis shift, in V1-4, and right ventricular strain pattern with T wave inversion.

**Figure 4:**
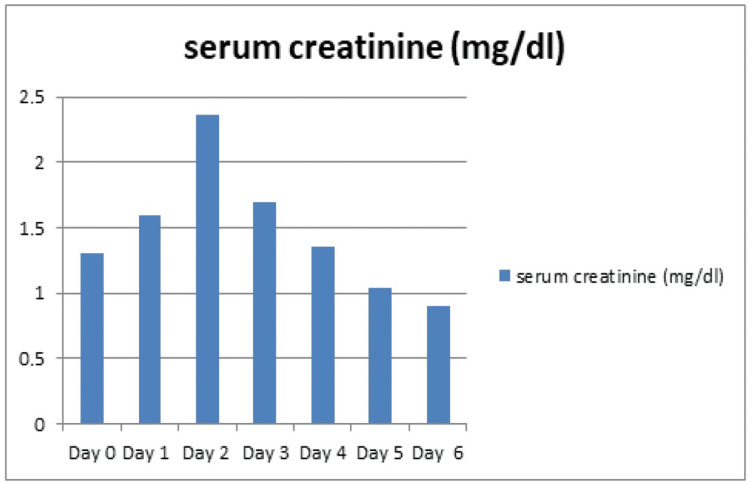
Time flow and creatinine course. The patient was admitted to the hospital on day 0. He was consulted on day two.

Electrocardiograms (ECGs) obtained on the day of consultation and the day the kidneys recovered are shown in Figure 2 and Figure 3, respectively. The cardiovascular examination was unremarkable. Based on a preliminary diagnosis of prerenal acute kidney injury, which was attributed to suspected extravasation from thrombophlebitis in the left lower extremity, the patient was started on intravenous fluids at 1500 mL/day on the first day of admission, which was continued for three days. On the fourth day, fluid administration was immediately discontinued, and diuretic therapy was initiated with close monitoring of his fluid balance. Calcium polystyrene sulfonate was administered for hyperkalemia, as there were no associated electrocardiographic changes. The patient’s urine output increased, and his dyspnea resolved, a clinical improvement that supported the continuation of diuretics. Over the following days, his renal function and hyperkalemia steadily improved (Figure 4). His treatment included unfractionated heparin for DVT and amoxicillin for suspected cellulitis. With the resolution of his dyspnea, improvement in mobility, and return of his appetite, the patient was discharged.

## Discussion

This case illustrates the clinical course of a patient who, despite lacking signs of volume overload on initial physical examination, developed worsening renal dysfunction and overt heart failure symptoms after receiving intravenous fluid resuscitation for a deep vein thrombosis and cellulitis. In the differential diagnosis for the patient's acute kidney injury, potential causes, such as nephrotoxic drug exposure, septic shock, overt signs of heart failure, hypotension, and hypoxemia, were not present. He was administered intravenous normal saline based on a preliminary diagnosis of prerenal acute kidney injury, presumed to be secondary to extravasation in the left lower extremity from cellulitis and deep vein thrombosis (DVT). This intervention likely increased renal venous congestion and thereby exacerbated the underlying renal impairment.

Cardiorenal syndrome (CRS) describes the process whereby dysfunction in either the heart or kidneys induces dysfunction in other organs [13]. For many years, the principal cause of renal dysfunction in heart failure was thought to be reduced renal arterial perfusion [1]. However, multiple studies and meta-analyses now indicate that elevated renal venous pressure, rather than decreased arterial perfusion, is the primary determinant of the decline in glomerular filtration rate in heart failure [1,5,14]. The pathophysiology of CRS involves a complex neurohormonal cascade. Hemodynamic compromise reduces the effective circulating volume, which stimulates the baroreceptors and activates systems such as the renin-angiotensin-aldosterone system [15]. The resultant sodium and water retention accumulates primarily in the venous vasculature, a compliant system that holds approximately 70% of the body's total blood volume [16]. A critical insight, however, is that acute decompensation is not merely a function of total volume gain but rather the result of rapid fluid redistribution. In compensated heart failure, a balanced venous reservoir capacitance (VRC) maintains hemodynamic equilibrium. When this state is disrupted for any reason, neurohormonal activation causes a rapid decrease in VRC, which shunts blood from the venous reservoir (particularly the splanchnic circulation) into the effective arterial circulation [17,18]. This fluid shift can be substantial and swift; studies showed that sympathetic activation can mobilize up to 800 mL of blood in seconds without any corresponding change in total body weight [17,19]. This rapid translocation of volume sharply increases ventricular filling pressures, precipitating acute decompensation and raising renal venous pressure even in the absence of clinically apparent hypervolemia [18,20]. This dynamic, driven by a sympathetically mediated loss of venous capacitance, explains how congestive nephropathy can develop without the classic signs of fluid overload [17,21]. Therefore, in patients with heart failure who are hospitalized for impaired renal function, congestive nephropathy should be considered in the differential diagnosis, even in the absence of overt signs of hypervolemia. Our patient's course aligns with this model, as the intravenous fluids administered to correct a presumed prerenal state instead worsened the underlying congestion and made the cardiorenal syndrome clinically manifest.

Nearly half of all patients hospitalized for heart failure are discharged before congestion is fully resolved [22]. Unresolved congestion prolongs hospitalization, increases readmission rates, and diminishes quality of life [22]. The presence of congestion in heart failure is associated with increased morbidity and mortality [23]. Because congestion affects multiple organ systems, its management requires a multidisciplinary approach that extends beyond cardiology [24]. Early recognition and prevention of congestion are critical, as a persistent state of congestion accelerates the progression of heart failure, promotes adverse cardiac remodeling and dilatation, and can lead to cardiac ischemia [24]. Breaking this vicious cycle at an early stage is crucial for improving patient prognosis. A study by Costanzo et al. demonstrated that early decongestive therapy, initiated before the development of overt congestion, reduces the length of hospital stay and the rate of readmission [25]. Furthermore, early decongestion facilitates the timely initiation of guideline-directed medical therapies, which are known to improve survival and, in turn, reduce readmissions, morbidity, and mortality [26].

For patients whose volume status is not clear on physical examination, point-of-care ultrasonography (POCUS) has emerged as a critical diagnostic tool. The venous excess ultrasound (VExUS) grading system, for example, allows for the objective quantification of systemic congestion by integrating the diameter of the inferior vena cava with Doppler waveforms from the hepatic, portal, and intrarenal veins [16,27]. The system is based on observing the abnormal Doppler waveforms that manifest in the inferior vena cava and downstream organs as a consequence of elevated central venous pressure (CVP) [28]. CVP rises when the mean filling pressure in the right heart exceeds the compliance of the right ventricle, a condition often caused by increased intravascular volume [28]. In a patient with stable cardiac function, administering 1000 mL of crystalloid fluid will initially increase preload and subsequently cardiac output, restoring hemodynamic balance. However, if the fluid load exceeds right ventricular compliance, CVP will rise rapidly, leading to congestion in downstream organs and the appearance of abnormal Doppler waveforms, which elevates the VExUS score [27,29]. An elevated VExUS score can also occur in other conditions, potentially leading to misinterpretation. For instance, in an acute pulmonary embolism, the rapid increase in right ventricular pressure causes an abrupt rise in CVP and VExUS grade [28]. Likewise, conditions, such as severe tricuspid regurgitation or high positive end-expiratory pressure (PEEP) in mechanically ventilated patients, can increase the VExUS score by raising CVP in the absence of hypervolemia [28]. A high VExUS score should be interpreted as evidence of organ congestion, regardless of the underlying cause; attributing it solely to hypervolemia can be misleading [28]. Acquiring an accurate VExUS score can be challenging, as it requires a capable Doppler ultrasound machine and a significant training period. Inexperience and equipment limitations can lead to inaccurate scores. Therefore, the VExUS score should always be interpreted in conjunction with the patient's physical examination and laboratory findings. In cases of discrepancy, the VExUS assessment should be re-evaluated.

In ambiguous cases, certain biomarkers can be utilized in addition to POCUS. B-type natriuretic peptide (BNP) and its N-terminal fragment, pro-BNP (NT-proBNP), are the most extensively studied natriuretic peptides for the diagnosis and prognosis of acute heart failure, and clinical guidelines recommend measuring NT-proBNP levels [22]. More recently, carbohydrate antigen 125 (CA-125), a biomarker traditionally used to monitor ovarian cancer, has also been reported to be elevated in acute heart failure [24]. It is hypothesized that increased intra-abdominal hydrostatic pressure stimulates CA-125 production by mesothelial cells, suggesting that CA-125 may serve as a useful biomarker for tissue congestion [7]. Other markers can also indicate organ-specific congestion. For example, hepatic congestion is often associated with elevated levels of total bilirubin, alkaline phosphatase (ALP), and gamma-glutamyl transferase (GGT) [24]. Similarly, acute renal congestion can cause glomerular and tubular dysfunction, resulting in mild albuminuria and tubular proteinuria [5]. Therefore, the mentioned laboratory parameters can be valuable in the differential diagnosis of congestive nephropathy.

In settings where POCUS is not available, a diagnostic intravenous fluid challenge may be considered [20]. After other causes of AKI are reasonably excluded, renal function is monitored closely following the administration of a small fluid bolus [9]. A paradoxical worsening of renal function strongly suggests underlying congestion, a finding that should prompt the immediate cessation of fluids and the initiation of diuretic therapy. The inadvertent fluid administration in our patient effectively served as a challenge; the resulting decline in renal function confirmed the diagnosis and led to the correct reversal of treatment, resulting in a subsequent rapid improvement in his creatinine levels.

Diuretics should be used with caution in patients with acute decompensated heart failure who present with hypotension. Such patients should be managed in an intensive care unit with close hemodynamic monitoring [30]. Upon presentation, disease-modifying therapies that may contribute to hypotension, such as angiotensin-converting enzyme (ACE) inhibitors, mineralocorticoid receptor antagonists (MRAs), and thiazide diuretics, should be temporarily discontinued or have their dosages reduced [31]. Positive inotropic and vasopressor agents should be initiated to support blood pressure [30]. Decongestive therapy can begin by administering an intravenous (IV) dose of a loop diuretic equivalent to the patient's oral home dose [30]. The subsequent dosage should be titrated based on the patient's response to the diuretic. Diuretic resistance should be suspected if the spot urine sodium is less than 50-70 mmol/L after 2 h or if the hourly urine output remains below 150 mL over 6 h. If blood pressure is stable, the loop diuretic dose should be doubled [31]. If an adequate response is still not achieved, combination therapy with a diuretic acting on a different nephron segment should be considered [30]. The loop diuretic dose can be doubled every 6 h until a target hourly urine output of 100-150 mL is achieved [30]. However, a total daily IV furosemide dose exceeding 600 mg is generally not recommended [32]. In refractory cases, ultrafiltration, for instance via continuous venovenous hemofiltration, should be considered [30].

Congestive nephropathy is frequently overlooked by clinicians. This is largely because the traditional pathophysiological explanation is as follows: in decompensated heart failure, it is believed that heightened neurohormonal and sympathetic activity causes vasoconstriction, thereby reducing renal perfusion pressure and eGFR. It is crucial to recognize that impaired renal function can also be driven by elevated renal venous pressure. Congestive nephropathy can develop even in the absence of overt signs of hypervolemia [17]. Adjunctive tools like POCUS are valuable for assessing this subclinical volume overload. Effective decongestion is known to reduce hospital readmissions and mortality [25]. Despite this, many patients with heart failure are discharged without achieving adequate decongestion, which contributes to increased morbidity and mortality [22]. It is also important to distinguish that "worsening renal function" in this context is not synonymous with the formal definition of acute kidney injury; it is typically a hemodynamic, functional, and reversible condition. Further studies are needed, and this concept should be carefully considered in the development of future clinical guidelines.

## Conclusions

Congestive nephropathy should be considered in the differential diagnosis for patients with heart failure and impaired renal function who present to the emergency department, regardless of the primary complaint. These patients may not have overt signs of hypervolemia. In ambiguous cases, POCUS should be used to assess for organ congestion, and decongestion should be achieved with diuretics or ultrafiltration. Prolonged organ congestion leads to structural damage, which increases morbidity and mortality. Therefore, patients require intermittent follow-up after discharge, with treatment adjustments made as needed to prevent recurrent congestion. While performing POCUS may be challenging for some clinicians, regular training can increase proficiency. Further research is also needed to develop simpler and equally reliable diagnostic methods. Finally, the distinction between "worsening renal function" and the formal definition of "acute kidney injury" in this patient population should be re-evaluated for future clinical guidelines, an area that also requires further investigation.
